# Chronic glucolipotoxic conditions in pancreatic islets impair insulin secretion due to dysregulated calcium dynamics, glucose responsiveness and mitochondrial activity

**DOI:** 10.1186/1471-2121-14-31

**Published:** 2013-07-01

**Authors:** Baggavalli P Somesh, Mahesh Kumar Verma, Manoj Kumar Sadasivuni, Anup Mammen-Oommen, Sanghamitra Biswas, Pavagada C Shilpa, Ashok Kumar Reddy, Aggunda N Yateesh, Puttrevana M Pallavi, Siddaraju Nethra, Rachapalli Smitha, Korrapati Neelima, Usha Narayanan, Madanahalli R Jagannath

**Affiliations:** 1 Connexios Life Sciences Pvt Ltd.,, No. 49, First Main road, 3rd phase, JP Nagar, Bangalore 560 078, India

**Keywords:** Type 2 diabetes, Rat islets, Glucolipotoxicity, Glucose metabolism, Insulin content, Insulin secretion

## Abstract

**Background:**

In the progression towards diabetes, glucolipotoxicity is one of the main causes of pancreatic beta cell pathology. The aim of this study was to examine the *in vitro* effects of chronic glucolipotoxic conditions on cellular responses in pancreatic islets, including glucose and fat metabolism, Calcium mobilization, insulin secretion and insulin content.

**Results:**

Exposure of islets to chronic glucolipotoxic conditions decreased glucose stimulated insulin secretion *in vitro.* Reduced protein levels of Glut2/slc2a2, and decreased glucokinase and pyruvate carboxylase mRNA levels indicated a significant lowering in glucose sensing. Concomitantly, both fatty acid uptake and triglyceride accumulation increased significantly while fatty acid oxidation decreased. This general suppression in glucose metabolism correlated well with a decrease in mitochondrial number and activity, reduction in cellular ATP content and dampening of the TCA cycle. Further, we also observed a decrease in IP3 levels and lower Calcium mobilization in response to glucose. Importantly, chronic glucolipotoxic conditions *in vitro* decreased insulin gene expression, insulin content, insulin granule docking (to the plasma membrane) and insulin secretion.

**Conclusions:**

Our results present an integrated view of the effects of chronic glucolipotoxic conditions on known and novel signaling events, *in vitro*, that results in reduced glucose responsiveness and insulin secretion.

## Background

Type 2 diabetes mellitus (T2DM) is a metabolic disorder in which pancreatic insulin secretion does not meet the demands of insulin sensitivity [1,2]. Over a period of time, consistently elevated levels of blood glucose and free fatty acids lead to glucolipotoxicity- mediated pancreatic beta cell dysfunction [3,4]. It is now accepted that elevated glucose levels are required to mediate the lipotoxic effects, including inhibition of glucose-stimulated insulin secretion (GSIS), impaired insulin gene expression and apoptosis [4-8].

GSIS involves both glucose oxidation-coupled ATP production and the anaplerotic/cataplerotic pathway-mediated generation of coupling factors that trigger and amplify insulin secretion, respectively [9,10]. Briefly, glucose uptake initiates metabolic pathways in which glucose is first converted to pyruvate mediated by glucokinase, and then to oxaloacetate by pyruvate carboxylase. Mitochondrial oxaloacetate generates citrate, a cataplerotic signal, which is transported to the cytosol and then broken down into acetyl-CoA initiating fatty acid synthesis. Acetyl-CoA is subsequently converted to malonyl-CoA, the concomitant step in fatty acid synthesis. In pancreatic beta cells, malonyl-CoA inhibits carnitine-palmitoyl transferase-1 (CPT-1) blocking fatty acid oxidation and resulting in the buildup of long-chain acyl-CoA esters (LC-CoA) in the cytosol [10]. Long chain-CoA is thought to be a potential modulator of insulin secretion stimulating insulin granule docking and exocytosis [11,12]. Glucose metabolism also raises the cytosolic ATP/ADP ratio, which inhibits the ATP-sensitive potassium channel (K_ATP_) resulting in plasma membrane depolarization. In response to this, voltage-gated calcium channels open, causing an influx of extracellular calcium and exocytosis of insulin granules [13].

Another well-known role of glucose is augmenting insulin secretion by promoting phospholipase-C (PLC)-mediated hydrolysis of phosphatidylinositol 4, 5-biphosphate (PIP2) into diacylglycerol (DAG) and inositol triphosphate (IP3) [14]. The DAG generated, in turn, activates protein kinase C (PKC), which is known to maintain insulin exocytosis [15,16], while IP3 mobilizes calcium from endoplasmic reticulum stores. The PLC pathway is also known to upregulate cAMP levels in beta cells, which show glucose-mediated oscillations that correlate with insulin secretion [17,18].

Further, glucose is known to increase insulin content through insulin gene transcription mediated by PDX1 and MAFa [19]. Under normal conditions, the synthesized insulin is held in readily releasable pools which are transported to the plasma membrane by the small GTPase, Rab27a and the SNARE complex for acute calcium-mediated release [20,21].

Chronic hyperglycemia (glucotoxicity) and hyperlipidemia (lipotoxicity) have been known to impair beta cell function [22,23], and glucolipotoxicity has been defined as ‘the deleterious effects of elevated glucose and fatty acids on pancreatic beta cell-function and mass’ [24]. Studies by Kashyap et al. in human subjects have shown that the ability of the beta cell to increase insulin secretion in response to fatty acids is a component that may predispose to T2DM [25]. In accordance with this, animal models for T2DM show a glucolipotoxicity-mediated dysfunction in multiple cellular processes involved in insulin secretion [26–27 and references therein]. *In vitro* studies have been an important source of information to understand the molecular basis of glucolipotoxicity. For example, fatty acid-mediated inhibition of insulin gene transcription, which was identified *in vitro,* has been recapitulated *in vivo*. However, a known limitation of the *in vitro* studies in this area of research has been the varying concentrations of fatty acid used [26].

Here, we used specific concentrations of glucose and palmitate to study the effects of *in vitro* chronic glucolipotoxic conditions on intracellular signaling pathways and cellular processes that mediate glucose responsiveness and insulin secretion. We confirmed metabolic stress in pancreatic islets under these conditions using known stress markers. We found that chronic glucolipotoxicity impaired glucose and fat uptake/metabolism in rat pancreatic cells resulting in lower cellular ATP along with mitochondrial number and activity. In agreement with this, IP3 levels were also reduced as was the calcium mobilized by the IP3 receptor and the L-type voltage gated calcium channels. Finally, we found that chronic glucolipotoxicity significantly decreased insulin secretion by reducing both insulin gene expression and granule docking to the plasma membrane in pancreatic islets. Thus, our results present the first integrated view of glucolipotoxicity *in vitro* linking known and novel signaling events to reduced glucose sensitivity and insulin secretion.

## Results

To investigate the effects of chronic glucolipotoxicity on glucose responsiveness and insulin secretion, we generated glucolipotoxic conditions in rat pancreatic islets and the NIT1 beta cell line using 16.7 mM glucose and 500 μM palmitate.

### Chronic glucolipotoxicity reduces insulin secretion in rat pancreatic islets

**Figure 1:**
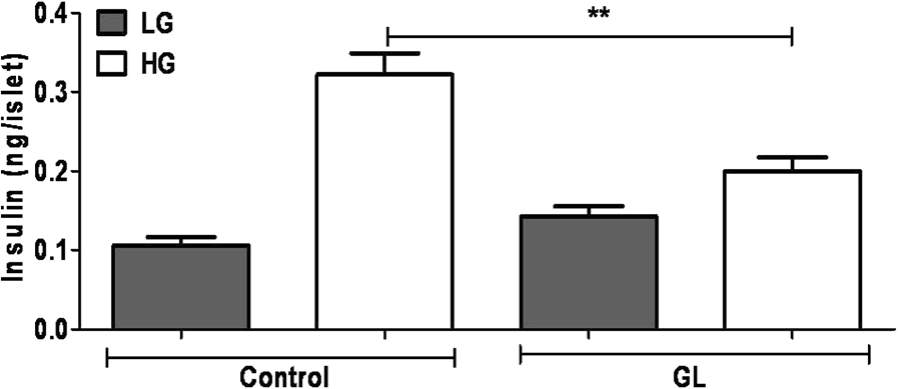
**Chronic glucolipotoxic conditions *in vitro *reduce GSIS.** Rat islets were cultured under normal conditions (control) or with 16.7 mM glucose and 500 μM palmitate for 72 h (GL). Post 72 h, islets were treated with 2 mM glucose (LG) or 11 mM glucose (HG) for 2 h and secreted insulin was measured. Data are expressed as mean±SEM and statistical analysis was performed using the unpaired Student’s t-test. (*P<0.05, **P<0.01 and ***P<0.001, n=4).

To evaluate the effect of high glucose and fatty acid concentrations on insulin secretion, we incubated rat pancreatic islets as mentzioned above for 72 h (chronic glucolipotoxic conditions that mimic diabetic pathology [26]); untreated islets were used as control. Under these conditions, we treated rat pancreatic islets with either low glucose or high glucose for 2 h to study glucose-stimulated insulin secretion (GSIS) (Figure 1). In agreement with previous studies [27], in the presence of high glucose, islet insulin secretion was significantly reduced under chronic glucolipotoxic conditions (Figure 1). We confirmed induction of glucolipotoxicity-mediated ER stress, oxidative stress and inflammation in pancreatic islets using known metabolic stress markers [28,29] (Additional file 1: Figure S1 and Additional file 2: Figure S2).

To understand the mechanism by which chronic glucolipotoxic conditions reduce GSIS *in vitro*, we next assessed glucose uptake/metabolism, calcium release, insulin gene expression and granule docking.

### Glucose uptake and metabolism is impaired under chronic glucolipotoxic conditions

**Figure 2:**
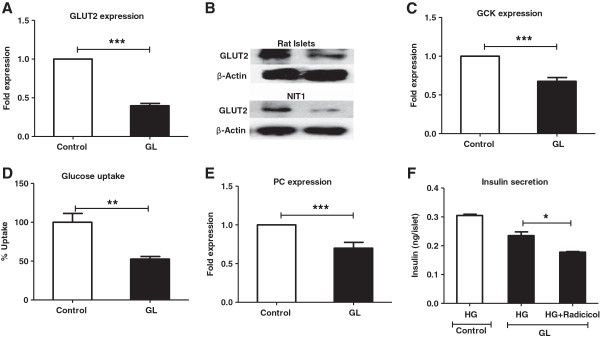
***In vitro *chronic glucolipotoxicity impairs glucose uptake/metabolism.** Rat islets or NIT1 cells were cultured under normal conditions (control) or with 16.7 mM glucose and 500 μM palmitate for 72 h (GL). Post 72 h, RNA was isolated from islets and the mRNA levels of Glut2/slc2a2 **(A),** GCK **(C)** and PC **(E)** were measured as described in the materials and methods. Protein levels of Glut2/slc2a2 **(B)** were measured in both islets and NIT-1 cells by western blotting using anti-Glut2/slc2a2 antibodies. Glucose uptake **(D)** was measured NIT-1 cells using 2-NBDG, a non-metabolized fluorescent analog as described in the materials and methods. **(F)** For insulin secretion in the presence of ATP citrate lyase (ACLY) inhibitor (Radicicol), post 72 h treatment islets were treated with 11 mM glucose (HG) with/without 50 μM inhibitor for 2 h and secreted insulin were measured. Data are expressed as mean±SEM and statistical analysis was performed using the unpaired Student’s t-test. (*P<0.05, **P<0.01 and ***P<0.001, n=4).

We examined the effect of chronic glucolipotoxic conditions on glucose metabolism in rat pancreatic islets and NIT1 cells maintaining the same experimental conditions used in Figure 1. Untreated rat islets and NIT1 cells, respectively were used as controls. We found significant reductions in the mRNA and protein levels of the glucose transporter, Glut2/Slc2a2 under chronic glucolipotoxic conditions when compared to the untreated control (Figure 2A and B) [30]. This reduction in Glut2 seen in both NIT-1 cells and rat pancreatic islets (Figure 2B) suggested impaired glucose metabolism, which was confirmed by a decrease in Glucokinase (Gck) and pyruvate carboxylase (Pc) mRNA levels (Figure 2C and E) [31-33]. To determine whether glucose uptake was also affected under chronic glucolipotoxic conditions, we used a fluorescent glucose analog, 2-NBDG to monitor glucose uptake. Under chronic glucolipotoxic conditions, we observed a ~40% decrease in glucose uptake (Figure 2D) indicating that both glucose uptake and metabolism were impaired. Consistent with these data, NADPH levels decreased and lactate release increased under chronic glucolipotoxic conditions (Additional file 3: Figure S3) confirming a dysfunction in glucose metabolism. The increase in lactate release also suggests that pryuvate, the end product of glycolysis, was converted into a non-oxidative metabolite indicating that glucose oxidation is severely impacted under chronic glucolipotoxic conditions.

We next ascertained the link between malonyl-CoA formation and insulin secretion under chronic glucolipotoxic conditions. To this end, we treated rat islets cultured in glucolipotoxic conditions with high glucose and found a decrease in insulin secretion, as expected. Interestingly, when ATP citrate lyase (ACLY) was inhibited using radicicol [34], insulin secretion decreased further suggesting that ACLY and potentially the anaplerotic/cataplerotic pathways are involved in the dysregulation seen in insulin secretion (Figure 2F). Together, these results suggest that chronic glucolipotoxicity impairs glucose uptake and metabolism and thus, insulin secretion.

### Chronic glucolipotoxicity impairs fatty acid uptake and metabolism

**Figure 3:**
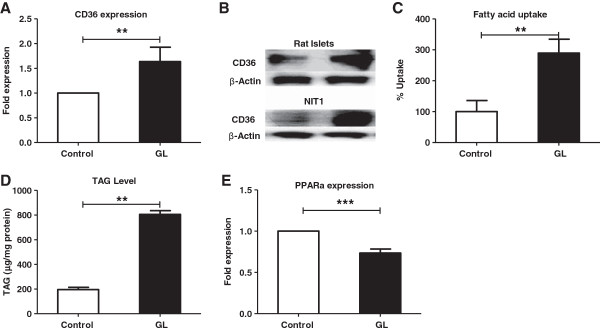
**Chronic glucolipotoxic conditions *in vitro *impair fatty acid uptake/ metabolism.** Rat islets and NIT1 cells were cultured under normal conditions (control) or with 16.7 mM glucose and 500 μM palmitate for 72 h (GL). Post 72 h, RNA was isolated from islets and the mRNA levels of cd36 **(A)** and PPARα **(E)** were measured as described in the materials and methods. Protein levels of CD36 in both islets and NIT-1 cells **(B)** were measured by western blotting using anti-CD36 antibodies. Fatty acid uptake **(C)** was measured in NIT-1 cells using green-fluorescent BODIPY dyes, a non-metabolized fluorescent labelled fatty acid analog as described in the materials and methods. For triglyceride estimation, post incubation cells were lysed and cellular triglyceride levels were estimated and normalized to total protein content **(D)**. Data are expressed as mean±SEM and statistical analysis was performed using the unpaired Student’s t-test. (*P<0.05, **P<0.01 and ***P<0.001, n=4).

Since chronic glucolipotoxic conditions impaired GSIS, we next investigated its effect on fatty acid metabolism [35]. We found that mRNA and protein levels of the fatty acid transporter, cd36 were significantly increased in rat islets (Figure 3A and B). This increase was observed in both NIT-1 cells and rat pancreatic islets (Figure 3B) suggesting increased fatty acid uptake.

To ascertain whether fatty acid uptake is impaired under chronic glucolipotoxic conditions, we used a BODIPY dye, a non-metabolized fluorescently labelled fatty acid analog. We observed a three-fold increase in fatty acid uptake under chronic glucolipotoxic conditions (Figure 3C) indicating that along with CD36 mRNA and protein levels, fatty acid uptake was also impaired. Further, we also found fat metabolism to be impaired under chronic glucolipotoxic conditions as seen from the four-fold increase in triglyceride levels in the pancreatic beta cell line, NIT-1 (Figure 3D). This was validated by a reduction in fatty acid oxidation studied by measuring the mRNA levels of PPARa (Figure 3E). We confirmed that *in vitro* chronic glucolipotoxicity generated metabolic stress in the cell system using known markers of ER stress [28,29] (Additional file 2: Figure S2A). Taken together, these data showed that chronic glucolipotoxic conditions impaired both glucose and fatty acid uptake and metabolism.

### Mitochondrial number/activity and cytosolic ATP levels are reduced under chronic glucolipotoxic conditions

**Figure 4:**
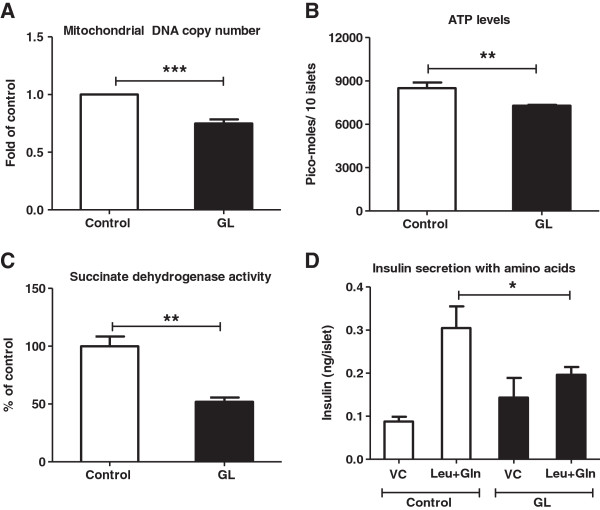
**Chronic glucolipotoxicity *in vitro *reduces mitochondrial number and activity.** Rat islets and NIT1 cells were cultured under normal conditions (control) or with 16.7 mM glucose and 500 μM palmitate for 72 h (GL). Post 72 h, total DNA was isolated from islets and mitochondrial DNA copy number **(A)** was estimated by assessing mtCox1 (mitochondrial gene) copy number normalized to HPRT (nuclear gene). Post treatment, islet ATP levels **(B)** were estimated after 60min induction with 11 mM (HG) as described in the materials and methods. Both SDH activity in NIT-1 cells **(C)** and amino acids (5 mM of leucine and 5 mM of glutamine) mediated insulin secretion **(D)** in islets were measured as described in the materials and methods. VC is vehicle control with 2 mM glucose and without amino acids. Data are expressed as mean±SEM and statistical analysis was performed using the unpaired Student’s t-test. (*P<0.05, **P<0.01 and ***P<0.001, n=4).

Since a primary outcome of glucose metabolism is ATP synthesis from mitochondria [27], we investigated the effect of chronic glucolipotoxic conditions on mitochondrial DNA copy number/activity and cellular ATP. Under chronic glucolipotoxic conditions, mtCox1 levels were significantly reduced in rat pancreatic islets along with a decrease in glucose-mediated cellular ATP (Figure 4A and B) suggesting a reduction in mitochondrial number. To ascertain the impact of decreased mtCox1 copy number on mitochondrial function under chronic glucolipotoxic conditions, we measured activity of succinate dehydrogenase- a critical enzyme in both the citric acid cycle and the mitochondrial respiratory chain. We found that under chronic glucolipotoxic conditions, succinate dehydrogenase activity decreased by ~50% (Figure 4C). This reduction in mitochondrial activity was further studied by measuring insulin secretion in the presence of leucine and glutamine, precursors of TCA cycle intermediates [10,36]. In this assay, chronic glucolipotoxic conditions reduced insulin secretion indicating an overall suppression of the TCA cycle (Figure 4D).

These data present the first line of evidence linking a decrease in cellular ATP to a reduction in mitochondrial number and activity under chronic glucolipotoxic conditions.

### An increase in cytoplasmic calcium is required for insulin secretion under chronic glucolipotoxic conditions

**Figure 5:**
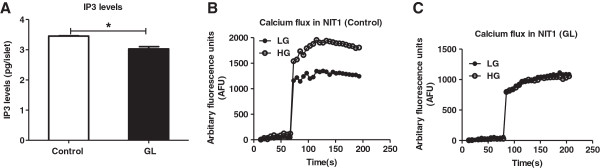
**Chronic glucolipotoxicity *in vitro *impairs calcium release.** Rat islets and NIT-1 cells were cultured under normal conditions (control) or with 16.7 mM glucose and 500 μM palmitate for 72 h (GL). Post 72 h, islet IP3 levels **(A)** were estimated in the presence of 11 mM (HG) as described in the materials and methods. For cytosolic calcium mobilization assay **(B and C)**, post treatment NIT-1 cells were loaded with Fluo-3AM calcium indicator dye for 1 h. After washing the cells, they were treated with either low (2.5 mM) or high (16.7 mM) glucose and the change in fluorescence was estimated as described in the materials and methods. Data are expressed as mean±SEM and statistical analysis was performed using the unpaired Student’s t-test. (*P<0.05, **P<0.01 and ***P<0.001, n=4).

Since chronic glucolipotoxicity lowered GSIS and glucose metabolism, we investigated its effects on IP3 and cytosolic calcium, known signaling mediators of insulin secretion [13,17]. We detected a modest decrease in IP3 upon culturing rat pancreatic islets under chronic glucolipotoxic conditions (Figure 5A). Next, we investigated intracellular calcium dynamics under chronic glucolipotoxic conditions in NIT-1 cells; cells cultured in 5mM glucose were used as control. Subsequently, cells were treated with either low or high glucose and cytosolic calcium was measured. In control cells, high glucose enhanced cytosolic calcium mobilization when compared to the low glucose treatment. Interestingly, this effect of high glucose on cytoplasmic calcium was lost under glucolipotoxic conditions (Figure 5B and C).

As further confirmation, we ascertained whether L-type voltage gated calcium channels mobilized calcium under glucolipotoxic conditions by studying insulin secretion in the presence or absence of the L-type channel inhibitor, Nitrendipine, NTD [37]. As reported earlier, we detected a decrease in high glucose-mediated secretion in the presence of NTD (Additional file 4: Figure S4A). In a similar assay, upon using the IP3 receptor inhibitor, 2-aminoethyldiphenyl borate (2-APB) [38], we found that endoplasmic reticulum calcium mobilization was also required for insulin secretion (Additional file 4: S4B). In summary, chronic glucolipotoxic conditions impaired IP3 levels and cytosolic calcium release.

### Insulin synthesis and intracellular insulin content are reduced under chronic glucolipotoxic conditions

**Figure 6:**
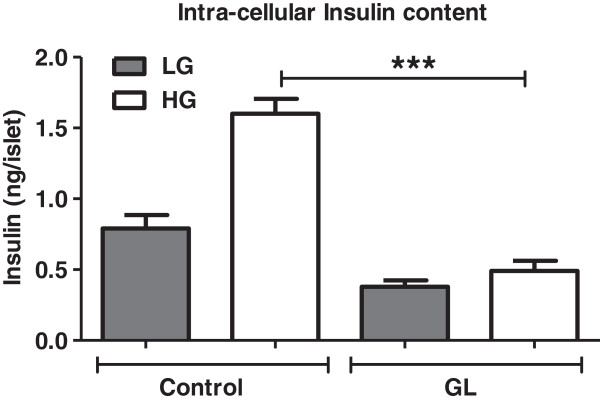
**Chronic glucolipotoxic conditions *in vitro *reduce intracellular insulin content.** Rat islets were cultured under normal conditions (control) or with 16.7mM glucose and 500 μM palmitate for 72 h (GL). Post 72 h, islets were treated with 2 mM glucose (LG) or 11 mM glucose (HG) for 2 h. Islets were washed, lysed and islet insulin content was measured. Data is expressed as mean±SEM and statistical analysis was performed using the unpaired Student’s t-test. (*P<0.05, **P<0.01 and ***P<0.001, n=4).

Calcium and cAMP are known to influence insulin gene expression through Pdx1 [39,40]. As reported previously [19], Pdx1 and insulin (Ins2) mRNA levels were reduced under chronic glucolipotoxic conditions (Additional file 5: Figure S5A and B). Thus, chronic glucolipotoxicity not only mediates its effects on GSIS by impairing glucose metabolism but also a down-regulation of insulin gene transcription. Lastly, we ascertained whether changes in insulin synthesis and glucose metabolism influenced insulin content (Figure 6). To test this, we treated rat islets (as in Figure 1) and observed a significant decrease in islet insulin content under chronic glucolipotoxic conditions (Figure 6). Thus, chronic glucolipotoxicity significantly affects overall glucose responsiveness through glucose metabolism, calcium release and insulin gene expression.

### Insulin granule docking is reduced under chronic glucolipotoxic conditions

In animal models of T2DM, the small GTPase, Rab27a is known to be downregulated leading to decreased insulin granule docking to the plasma membrane, thereby lowering insulin secretion [41]. In addition, insulin release at the fusion pore is also known to be impaired in diabetic animal models resulting in defects in exocytosis and release [42].

**Figure 7:**
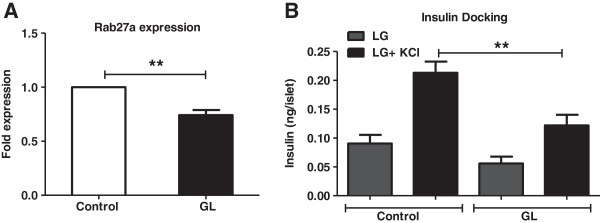
***In vitro *chronic glucolipotoxicity reduces insulin docking and exocytosis.** Rat islets were cultured under normal conditions (control) or with 16.7 mM glucose and 500 μM palmitate for 72 h (GL). Post 72 h, RNA was isolated from islets and the mRNA levels of Rab27a **(A)** were measured as described in the materials and methods. For measurement of insulin granule docking **(B)** following incubation under chronic glucolipotoxic conditions, islets were treated with LG (2.5 mM glucose) with or without 30 mM KCl for 30 min. Secreted insulin was measured as described in the materials and methods. Data are expressed as mean±SEM and statistical analysis was performed using the unpaired Student’s t-test. (*P<0.05, **P<0.01 and ***P<0.001, n=4).

Here, we found that Rab27a mRNA significantly decreased under chronic glucolipotoxic conditions in both pancreatic islets and NIT1 cells (Figure 7A and Additional file 5: Figure S5C) suggesting a decrease in the readily releasable pool of insulin granules docked to the plasma membrane. To confirm this, we treated rat islets with potassium chloride (KCl), a membrane-depolarizing agent known to release insulin vesicles docked to the plasma membrane [43]. As expected, we found a significant decrease in docked insulin granules under chronic glucolipotoxic conditions when compared to control islets (Figure 7B). These data show that chronic glucolipotoxicity reduces insulin secretion through its effects on insulin synthesis and transport in addition to glucose uptake/metabolism and cystosolic calcium mobilization.

Taken together, these data provide the first, integrated *in vitro* view of known dysfunctional cellular mechanisms in chronic glucolipotoxic conditions, while identifying novel events such as the glucolipotoxicity-mediated reduction in mitochondrial number/activity and insulin granule docking/transport.

## Discussion

Despite intensive research, information about the mechanism of action and intracellular signaling pathways activated by glucolipotoxicity remains limited. Such an understanding has clinical relevance since the ability of the beta cell to increase insulin secretion in response to fatty acids is thought to be a predisposing factor for T2DM [25]. *In vitro* studies have been important to gain a mechanistic understanding of glucolipotoxicity but have not allowed a complete view of glucolipotoxicity-mediated cellular dysregulation due to variations in the concentrations of fatty acids used [24]. This study systematically evaluates specific *in vitro* glucolipotoxic conditions linking their effect to multiple cellular processes involved in insulin secretion and glucose responsiveness including glucose uptake/metabolism, fatty acid uptake/metabolism, cellular energetics, insulin synthesis, secretion and transport; and calcium dynamics.

We used 16.7 mM glucose and 500 μM palmitate in this study after evaluating multiple concentrations for their effect on metabolic stress and cell death (unpublished observations). Under these conditions, we confirmed metabolic stress in pancreatic islets and the NIT1 pancreatic beta cell line as seen by the strong induction of ER stress, oxidative stress and inflammation. As previously reported [27], these conditions also led to cell death as seen by the significant increase in caspase-3 activity (Additional file 2: Figure S2B).

Under chronic glucolipotoxic conditions *in vitro*, we found that insulin content and GSIS were lowered in rat pancreatic islets. Further, glucose and fat metabolism were impaired in islets correlating with the decrease in mitochondrial number/activity and cellular ATP levels. Chronic glucolipotoxicity reduced cytosolic calcium levels by decreasing calcium mobilization mediated by ITPR.

**Figure 8:**
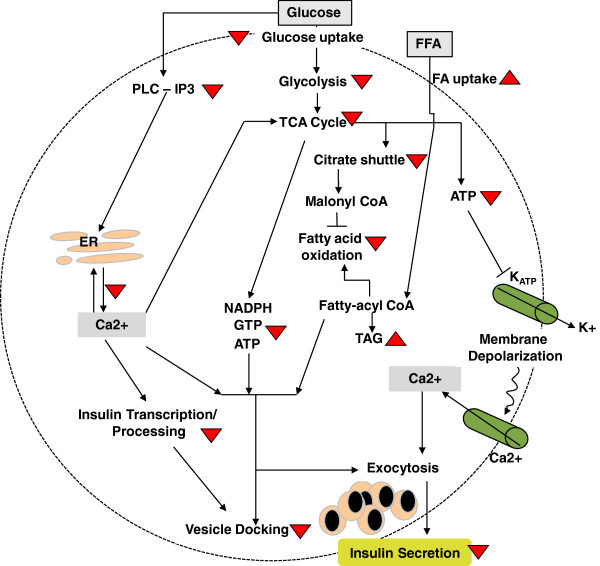
**Model for impact of *in vitro *chronic glucolipotoxicity on cellular processes in pancreatic beta cells.** Model describes the signaling pathways involved in glucose and fat metabolism with focus on their coupling to insulin secretion. We found that under chronic glucolipotoxic conditions *in vitro,* multiple signaling events involved in glucose and fat metabolism are dysregulated leading to impaired insulin synthesis, content and secretion. The effects of chronic glucolipotoxic conditions on the signaling events involved are shown using upward arrowheads (representing an increase) and downward arrowheads (representing a decrease). In the model, arrows indicate activation and blunt arrows indicate inhibition.

Based on our findings, we propose a model for the effect of chronic glucolipotoxicity on the pancreatic beta cell (Figure 8). Extended exposure to high glucose and palmitate concentrations leads to a) suppression of glycolysis resulting in reduced cellular ATP and a dampening of the TCA cycle, b) reduction in mitochondrial DNA copy number and activity, c) reduction in PLC-IP3 signaling leading to reduced calcium mobilization, insulin transcription and granule docking, and d) a decrease in insulin transcription and synthesis.

Our *in vitro* findings recapitulated data from previous glucolipotoxic studies in animal models showing an impact on glucose metabolism, calcium dynamics and insulin secretion/content [24,44]. For example, we detected an increase in CD36 expression under glucolipotoxic conditions that correlated with enhanced triglyceride accumulation and reduced GSIS. These findings concur with data from cd36-null mouse models and over-expression studies in INS cell lines [45,46] further validating the *in vitro* conditions used in our study.

We found it interesting that chronic glucolipotoxic conditions impacted multiple cellular processes including insulin synthesis, content and docking. On the basis of our results, we speculate that chronic glucolipotoxicity impacts insulin content most severely compared to insulin gene transcription, docking and secretion. Future studies will be required to get a more complete understanding of the same.

A key finding in our study is the influence of glucolipotoxicity on mitochondrial number/function. This is in line with the notion that enhanced insulin secretion may require an overall increase in mitochondrial activity/number as opposed to an isolated increase in an aspect of mitochondrial metabolism [47,48]. In this study, we also detected a decrease in insulin granule docking/release under glucolipotoxic conditions indicative of a reduction in the readily releasable pool of insulin, which may have a bearing on the first-phase insulin secretion [43]. Further studies are required to explore the mechanistic link between docking/exocytosis and the DAG-PKC pathway in more detail.

Increasing insulin secretion is an intensely pursued therapeutic strategy in T2DM. This study yields *in vitro* assay conditions that can be used to evaluate anti-diabetic agents, specifically insulin secretagogues, currently in development for their impact on glucolipotoxicity-mediated dysregulation. Importantly, an understanding of glucolipotoxicity-mediated cellular dysfunction may yield novel points of therapeutic intervention (i.e., targets/target classes) that hold promise in T2DM treatment. Thus, our study has potential to facilitate an improved understanding of pancreatic beta cell pathophysiology in T2DM.

## Conclusions

Chronic glucolipotoxic conditions comprising high glucose and fatty acid resulted in various defects in key cellular machineries. Glucose sensing machinery involved in uptake and glucose metabolism for insulin secretion was reduced whereas fat uptake and triglyceride storage was increased. Defects in mitochondrial number and activity along with reduced ATP levels were observed under glucolipotoxic conditions. Similarly, beta cells showed increased ER stress, inflammation and apoptosis together with impaired calcium homeostasis. These defects occurred in conjunction with decreased insulin synthesis, insulin vesicle transport, docking and glucose-dependent insulin secretion. Our data provide a first integrated view of beta cell defects across multiple levels under chronic glucolipotoxic conditions.

## Methods

### RNA isolation, reverse transcription and quantitative real time polymerase chain reaction (qPCR)

Isolation and preparation of rat islets has been described in detail in Additional file 6 (supplementary methods). All animal studies and protocols were approved by the Institutional Animal Ethics Committee (IAEC) of Connexios Life Sciences Pvt Ltd. Post 72 h of incubation, total RNA was isolated and 1 μg of total RNA was used to generate cDNA (ABI, USA). Gene expression was measured using SYBR Green PCR Master Mix (Eurogenetic, Belgium). Gene primers for Slc2a2/Glut2, Gck, Pc, CD36, PPARα, Pdx1, Ins2, Rab27a, Il1β, Nos2a and Actb were based on mRNA sequences from the GenBank nucleotide database and designed in-house. Actb was used as an internal control. The primer sequence for the above gene markers are given in the Additional file 6 (supplementary methods).

### Insulin secretion and content

Islets were isolated from rats (details in Additional file 6) and cultured in 90 mm petri-plates with RPMI 11 mM glucose and 10% FBS and penicillin streptomycin, in the presence or absence of 16.7 mM glucose and 500 μM palmitate for 72 h/37°C/5% CO2. Size-matched islets were isolated and transferred into 24-well plates containing 1ml KRBH (2.5 mM glucose)/well, and incubated at 37°C/5% CO2 for 1h. After removing the KRBH buffer, the islets were induced in KRBH buffer (250μl/well) at 37°C/5% CO2 for 2 h at indicated glucose concentrations with/without the specified pharmacological inhibitors. Inhibitors were used at the following concentrations: Radicicol (50 μM; Sigma) and Nitrendipine (5 μM; Sigma); 2-APB (10 μM, Sigma). Secreted insulin was measured in KRBH buffer using ELISA (Mercodia) as per manufacturer’s instructions. To measure insulin secretion in the presence of TCA cycle precursors, islets were prepared as above and treated with 5 mM leucine and 5 mM glutamine-containing KRBH for 2 h; 2 mM glucose without amino acids was used as a control. Islet lysates were used to measure intracellular insulin content and insulin levels were presented as ng insulin/islet.

### Western blotting

NIT1 (ATCC) cells or rat islets were cultured with 5.5 mM glucose (control) with or without 16.7 mM glucose and 500 μM palmitate (GL) for 72 h. After incubation, cells or islets were lysed and total proteins were resolved by SDS-PAGE followed by transfer to nitrocellulose membrane. Protein expression and phosphorylation was measured using

Antibodies against Glut2, CD36 (Abcam), BiP, CHOP, p-eIF2a or β-actin (Cell Signaling Technology) and HRP conjugated secondary antibody (Bio-Rad). The protein specific signals were detected using chemiluminescence substrate (Pierce) and were quantified using Image-J software (NIH).

### Measurement of glucose uptake

NIT1 cells were cultured with 5.5 mM glucose (control) with or without 16.7 mM glucose and 500 μM palmitate (GL) for 72 h. Post 72 h, cells were washed and incubated in glucose-free medium at 37°C for 30 min followed by incubation with 50 μM of 2-NBDG (2-(N-(7-Nitrobenz-2-oxa-1,3-diazol-4-yl)Amino)-2-Deoxyglucose; Invitrogen) for 15min. After lysis, 2-NBDG uptake was measured at 465 nm excitation/540 nm emission, and normalized to total cellular DNA as measured using bis-benzamide at 360 nm.

### Measurement of fatty acid uptake

NIT1 cells were cultured with 5.5 mM glucose (control) with or without 16.7 mM glucose and 500 μM palmitate (GL) for 72 h. Post 72 h, cells were washed and incubated in glucose-free medium at 37°C for 30 min followed by incubation with 1 μM of green-fluorescent BODIPY dyes (Molecular Probes) for 10 min. Cells were washed and incubated with 0.4% trypan blue for 5 min to quench any excess dye. Subsequently, cells were washed, lysed and BODIPY uptake was measured at 485 nm excitation/528 nm emission and normalized to total cellular proteins as measured using the Bradford assay (Bio-Rad). BODIPY uptake was represented as % of uptake under control condition.

### Estimation of triglycerides

NIT1 cells were cultured with 5.5 mM glucose (control) with or without 16.7 mM glucose and 500 μM palmitate (GL) for 72 h. After incubation, cells were washed with PBS and lysed. Total cellular protein was estimated using the Bradford assay (Bio-Rad) and triglyceride levels were estimated using an enzymatic assay (DiaSys) as per manufacturer’s instructions. TAG levels were normalized to cellular protein levels.

### Estimation of mitochondrial DNA copy number

Freshly isolated rat islets were cultured under control or glucolipotoxic conditions for 72 h. Post 72 h treatment, islets were harvested in digestion buffer (Tris-10mM, pH8.0, EDTA-1 mM, NaCl-5 mM, SDS-1% and RNAse-A-10 mg/ml) followed by a phenol-chloroform extraction and ethanol precipitation of total DNA. 5 μg DNA was used for quantitative real time PCR. Mitochondrial cytochrome C oxidase 1 (mtCox1) copy number was measured and normalized to nuclear DNA using hypoxanthine guanine phosphoribosyltransferase (HPRT).

### Measurement of islet ATP

Rat islets were cultured as in the insulin secretion assay. Islets were incubated in the KRBH buffer containing 2.5 mM glucose for 1h followed by induction with 11 mM glucose (HG) for 1h. After 1 h of incubation, islets were lysed and ATP levels were estimated as per manufacturer’s instructions (ATP determination kit, Invitrogen).

### Measurement of succinate dehydrogenase activity

NIT1 cells were cultured in 5.5 mM glucose (control) with or without 16.7 mM glucose and 500 μM palmitate (GL) for 72 h. After incubation, cells were washed and incubated in 100 mM potassium phosphate buffer containing 50 mM sucrose, 10 mM sodium azide, 500 mM sodium succinate and 8 mM INT (Iodonitrotetrazolium chloride; Sigma) for 2 h. Cells without sodium succinate were used as a negative control. After 2 h at 37°C, INT was dissolved in DMSO and estimated at 644 nm. The difference in absorbance with/ without succinate was calculated, normalized to total cellular protein and represented as % control SDH activity.

### Estimation of islet IP3

Freshly isolated rat islets were cultured under normal condition (control) or under glucolipotoxic condition (GL) for 72 h. Islets were then washed and incubated in KRBH containing 2.5 mM glucose for 1h followed by treatment with HG for 5 min. IP3 levels were measured in the lysate using an immunoassay kit (Cusabio IP3 estimation kit).

### Estimation of calcium mobilization

NIT1 cells were cultured with 5.5 mM glucose (control) with or without 16.7 mM glucose and 500 μM palmitate (glucolipotoxic) for 72 h. After incubation, cells were washed with calcium-free KRBH buffer followed by incubation at 37°C for 1 h in Fluo-3-AM calcium indicator fluorescent dye (Invitrogen). Cells were then induced with either low (2.5 mM) or high (16.7 mM) glucose and the fluorescence was measured at 485 nm. The baseline reading was established by reading fluorescence for 1 minute (from t=0 to t=1 minute) at 6 s intervals. The indicated glucose concentrations were added at t=1 minute. After mixing for 5 s, the final reading was taken for 3 minutes (from t=1 minute to t=4 min) at 6 s intervals.

### Exocytosis of docked insulin granules

Islets were cultured as in the insulin secretion assay, washed and incubated in KRBH buffer containing 2.5 mM glucose for 1h. Islets were treated with low glucose alone (2 mM) with/without 30 mM KCl in KRBH for 30 min followed by estimation of secreted insulin in the buffer.

### Statistical methods

Data are expressed as mean±SEM and significance was calculated using the unpaired Student’s t-test. * indicates p<0.05; ** indicates p<0.01; *** indicates p<0.001 compared to the respective control. Unless mentioned otherwise, n=4 across experiments. Miscrosoft Excel was used for statistical analyses.

## Competing interest

The authors do not declare any competing interest.

## Authors’ contributions

BS, RA, YAN, PPM, SN, PCS, RS, KN, SMK and VMK carried out experiments; VMK, SBP and JMR planned/executed the study and analyzed data. NU, MOA, VMK, SMK, SBP and JMR analyzed data and contributed to writing the paper. All authors read and approved the final manuscript.

## Supplementary Material

Additional file 1: Figure S1 Chronic glucolipotoxic conditions increase inflammatory cytokines gene expression and cellular stress in pancreatic β-cells. Rat islets or NIT1 cells were cultured under normal conditions (control) or with 16.7 mM glucose and 500 μM palmitate for 72 h (GL). Post 72 h, RNA was isolated from islets and the mRNA levels of IL1b **(A)** and Nos2a **(B)** were measured as described in the materials and methods. Both ROS **(C)** and nitric oxide **(D)** levels were estimated in NIT1 cells as described in the Additional file 6. Data are expressed as mean±SEM and statistical analysis was performed using the unpaired Student’s t-test. (*P<0.05, **P<0.01 and ***P<0.001, n=4). Click here for file

Additional file 2: Figure S2 Chronic glucolipotoxic conditions increase endoplasmic reticulum stress and apoptosis in pancreatic β-cells**.** NIT1 cells were cultured under normal conditions (control) or with 16.7 mM glucose and 500 μM palmitate for 72 h (GL). Post 72 h, changes in ER stress markers **(A)** were analyzed using anti-CHOP, anti-BiP and phospho-specific EIF2a antibodies; β-actin was used as the internal control. Both Caspase-3 activity **(B)** and cell viability (MTT assay) were measured in NIT1 cells as described in the Additional file 6. Data are expressed as mean±SEM and statistical analysis was performed using the unpaired Student’s t-test. (*P<0.05, **P<0.01 and ***P<0.001, n=4). Click here for file

Additional file 3: Figure S3 Glucose metabolism is impaired under glucolipotoxic condition. NIT1 cells were cultured under normal conditions (control) or with 16.7 mM glucose and 500μM palmitate for 72 h (GL). Post 72 h, cells were lysed and cellular NADPH levels were measured **(A)** and culture medium was used for the estimation of lactate levels **(B)**. Data are expressed as mean±SEM and statistical analysis was performed using the unpaired Student’s t-test. (*P<0.05, **P<0.01 and ***P<0.001, n=3-4). Click here for file

Additional file 4: Figure S4 Cytoplasmic calcium increase is required for insulin secretion under glucolipotoxic condition. Rat islets were cultured under normal conditions (control) or with 16.7 mM glucose and 500 μM palmitate for 72 h (GL). Post 72 h, islets were treated with 11 mM glucose (HG) alone or with inhibitors (5 μM Nitrendipine and 10 μM 2-APB) for 2 h and secreted insulin was measured as described in the materials and methods. Data are expressed as mean±SEM and statistical analysis was performed using the unpaired Student’s t-test. (*P<0.05, **P<0.01 and ***P<0.001, n=4). Click here for file

Additional file 5: Figure S5 Chronic GL conditions reduce insulin synthesis and vesicle transport. Rat islets or NIT1 cells were cultured under normal conditions (control) or with 16.7 mM glucose and 500 μM palmitate for 72 h (GL). Post 72 h, RNA was isolated from both islets and cells. The mRNA levels of PDX1 **(A)** and Insulin **(B)** were measured in islets and Rab27a levels **(C)** in cells were measured as described in the materials and methods. Data are expressed as mean±SEM and statistical analysis was performed using the unpaired Student’s t-test. (*P<0.05, **P<0.01 and ***P<0.001, n=4). Click here for file

Additional file 6 Supplementary methods. Click here for file
