# Supplementary figures and images for: Chronic glucolipotoxic conditions in pancreatic islets impair insulin secretion due to dysregulated calcium dynamics, glucose responsiveness and mitochondrial activity
Source: BMC Cell Biol. 2013 Jul 1;14:31. doi: 10.1186/1471-2121-14-31 (PMC3704974; doi:10.1186/1471-2121-14-31)

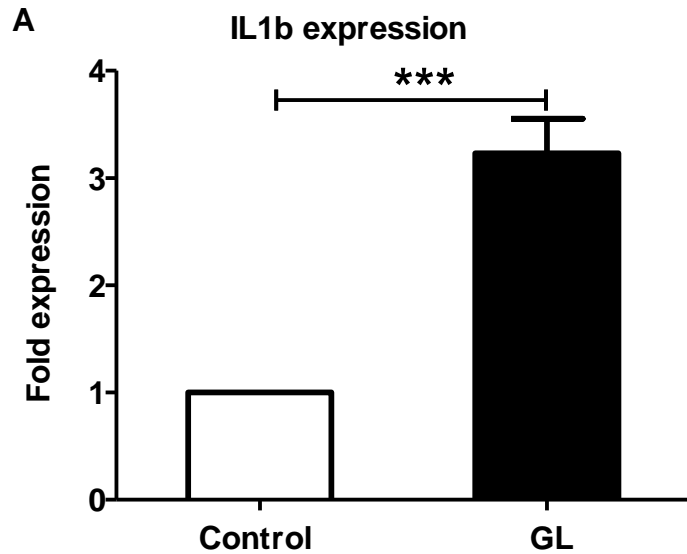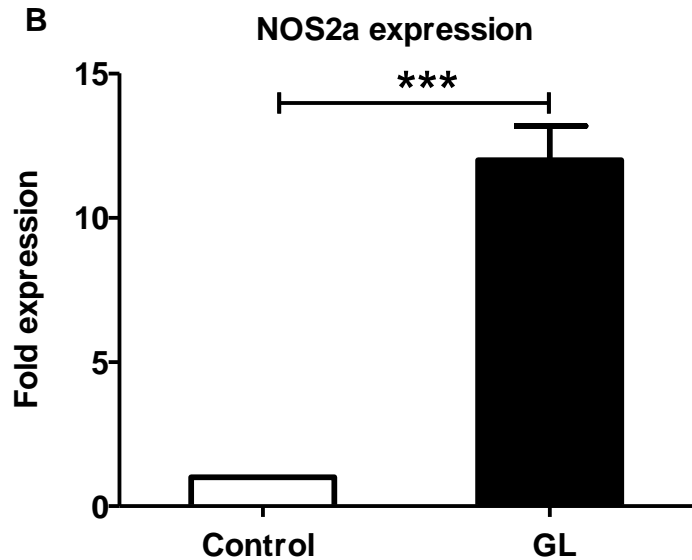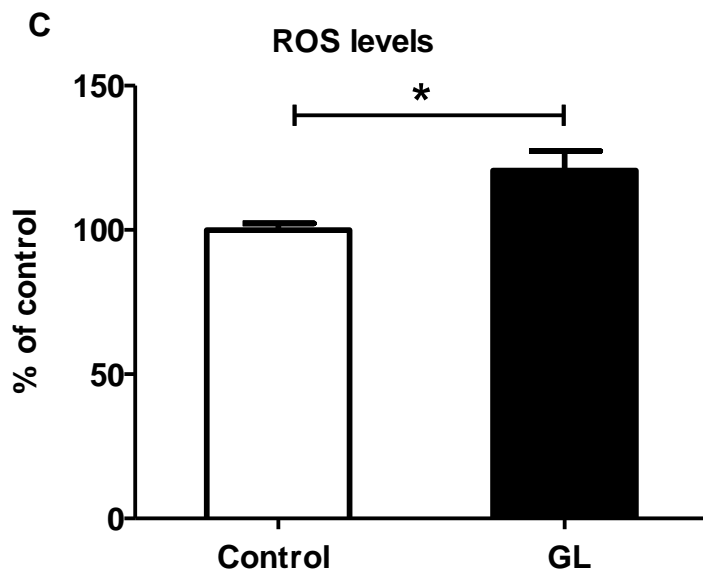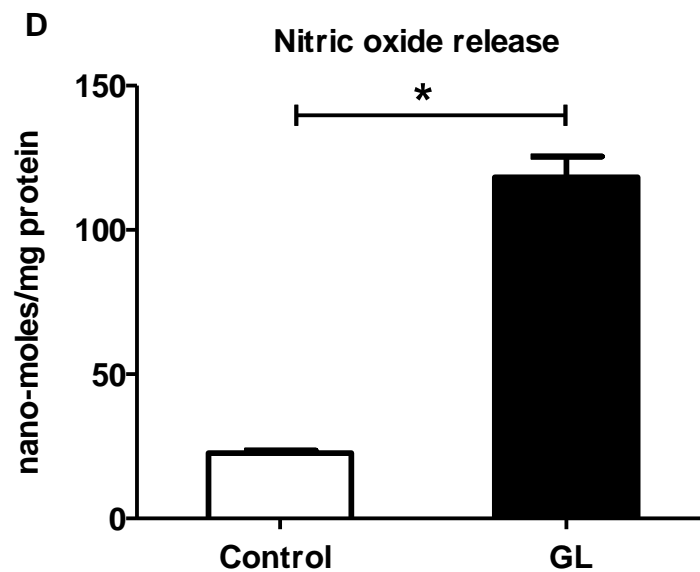

Supplement: Additional file 1: Figure S1 — Chronic glucolipotoxic conditions increase inflammatory cytokines gene expression and cellular stress in pancreatic β-cells. Rat islets or NIT1 cells were cultured under normal conditions (control) or with 16.7 mM glucose and 500 μM palmitate for 72 h (GL). Post 72 h, RNA was isolated from islets and the mRNA levels of IL1b (A) and Nos2a (B) were measured as described in the materials and methods. Both ROS (C) and nitric oxide (D) levels were estimated in NIT1 cells as described in the Additional file 6. Data are expressed as mean±SEM and statistical analysis was performed using the unpaired Student’s t-test. (*P<0.05, **P<0.01 and ***P<0.001, n=4). [file 1471-2121-14-31-S1.pdf]

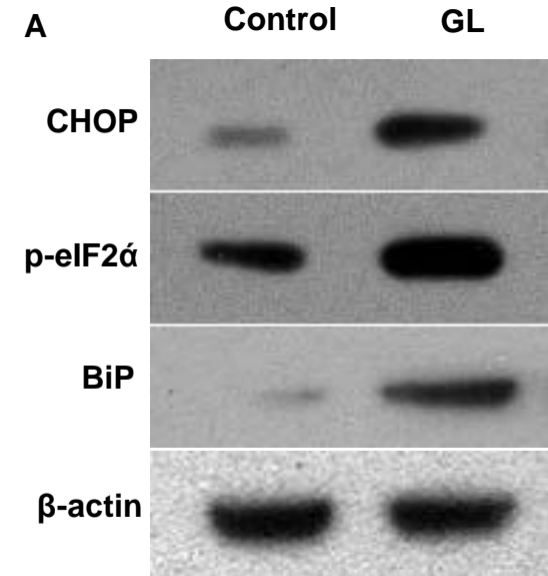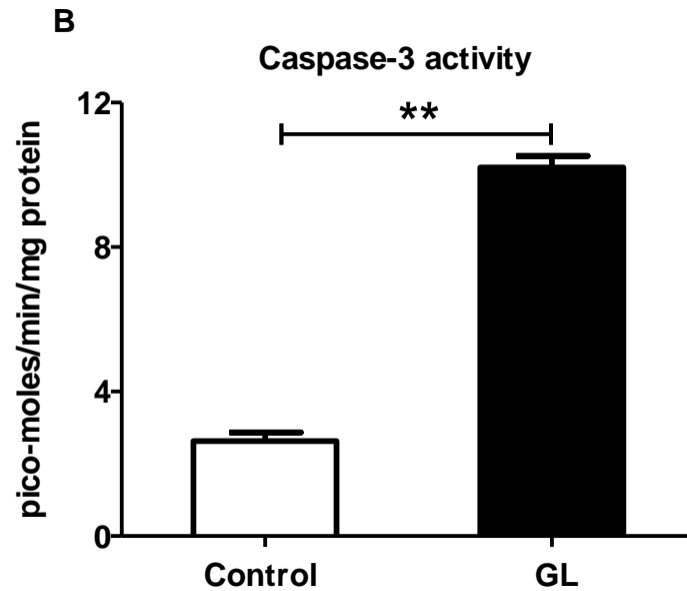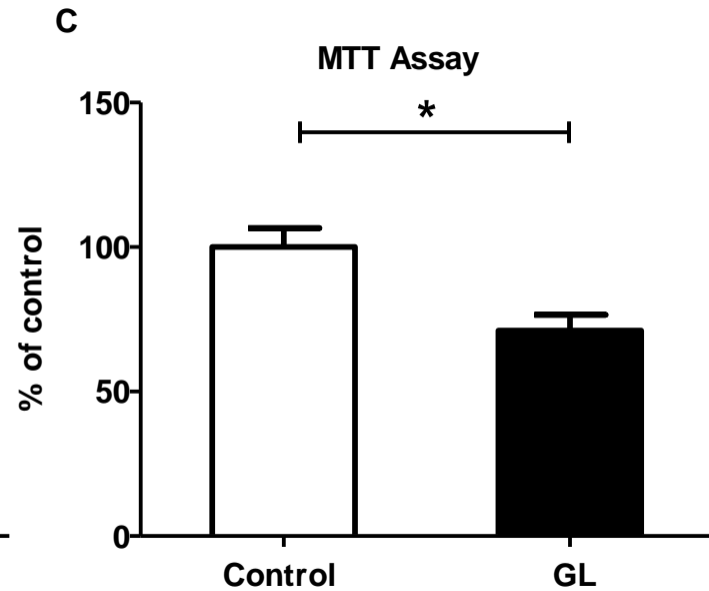

Supplement: Additional file 2: Figure S2 — Chronic glucolipotoxic conditions increase endoplasmic reticulum stress and apoptosis in pancreatic β-cells. NIT1 cells were cultured under normal conditions (control) or with 16.7 mM glucose and 500 μM palmitate for 72 h (GL). Post 72 h, changes in ER stress markers (A) were analyzed using anti-CHOP, anti-BiP and phospho-specific EIF2a antibodies; β-actin was used as the internal control. Both Caspase-3 activity (B) and cell viability (MTT assay) were measured in NIT1 cells as described in the Additional file 6. Data are expressed as mean±SEM and statistical analysis was performed using the unpaired Student’s t-test. (*P<0.05, **P<0.01 and ***P<0.001, n=4). [file 1471-2121-14-31-S2.pdf]

**A**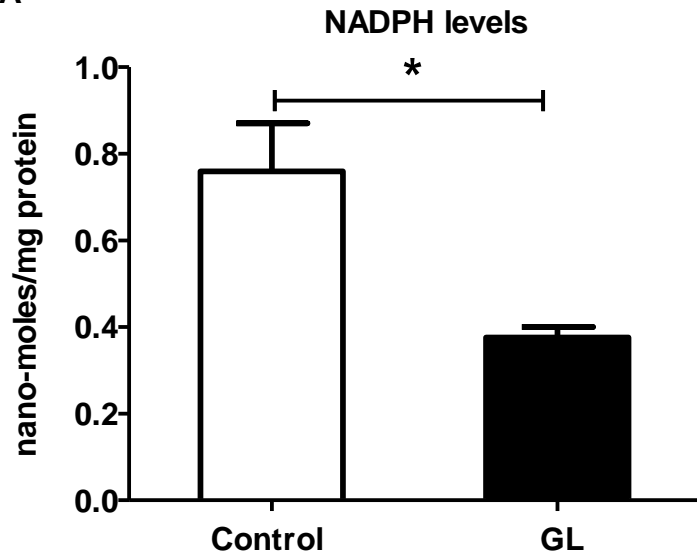**B**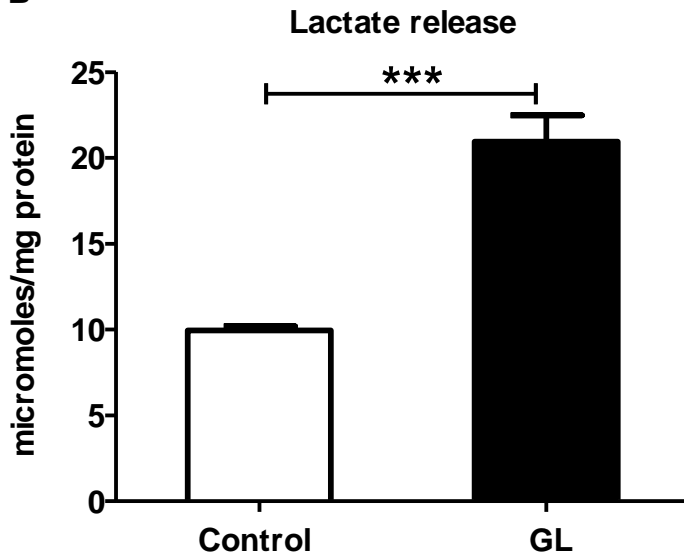

Supplement: Additional file 3: Figure S3 — Glucose metabolism is impaired under glucolipotoxic condition. NIT1 cells were cultured under normal conditions (control) or with 16.7 mM glucose and 500μM palmitate for 72 h (GL). Post 72 h, cells were lysed and cellular NADPH levels were measured (A) and culture medium was used for the estimation of lactate levels (B). Data are expressed as mean±SEM and statistical analysis was performed using the unpaired Student’s t-test. (*P<0.05, **P<0.01 and ***P<0.001, n=3-4). [file 1471-2121-14-31-S3.pdf]

**A**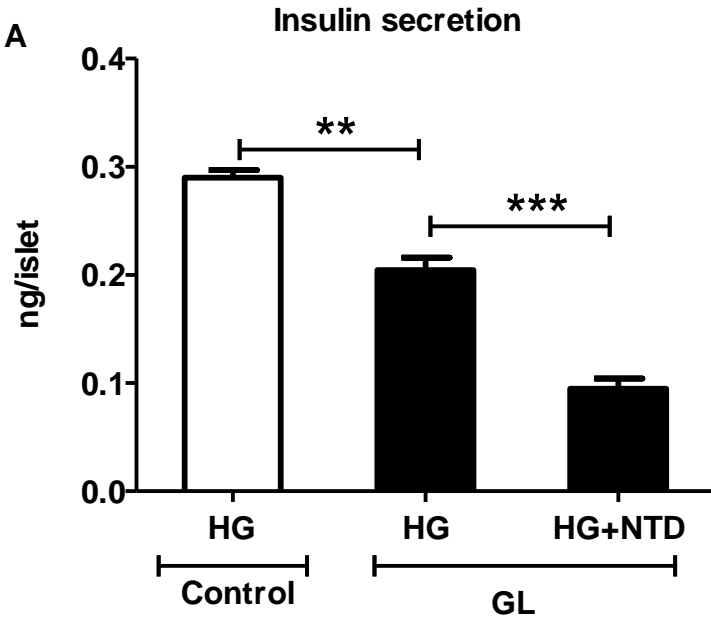**B**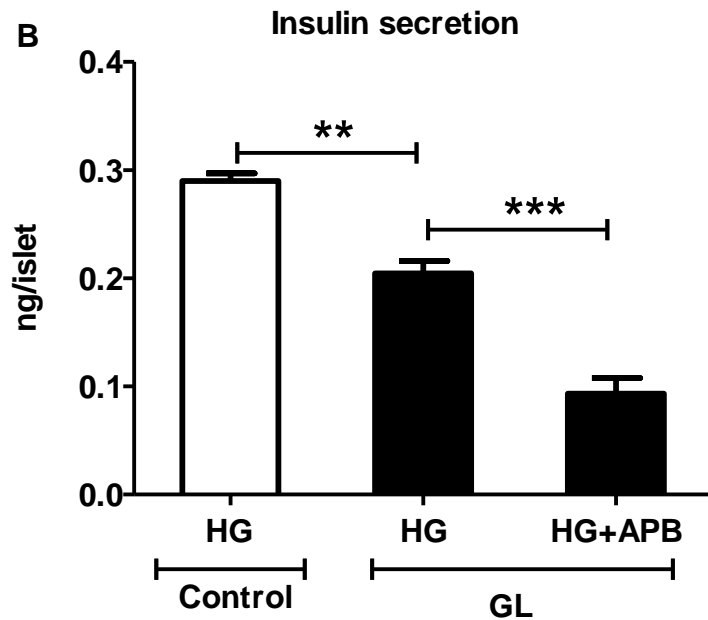

Supplement: Additional file 4: Figure S4 — Cytoplasmic calcium increase is required for insulin secretion under glucolipotoxic condition. Rat islets were cultured under normal conditions (control) or with 16.7 mM glucose and 500 μM palmitate for 72 h (GL). Post 72 h, islets were treated with 11 mM glucose (HG) alone or with inhibitors (5 μM Nitrendipine and 10 μM 2-APB) for 2 h and secreted insulin was measured as described in the materials and methods. Data are expressed as mean±SEM and statistical analysis was performed using the unpaired Student’s t-test. (*P<0.05, **P<0.01 and ***P<0.001, n=4). [file 1471-2121-14-31-S4.pdf]

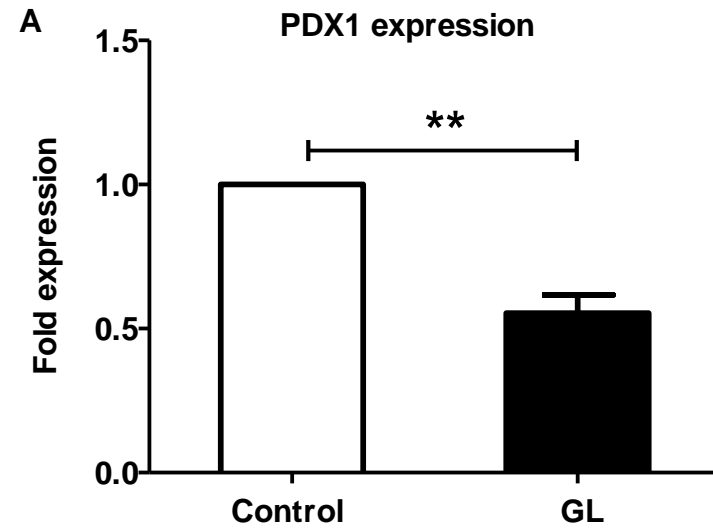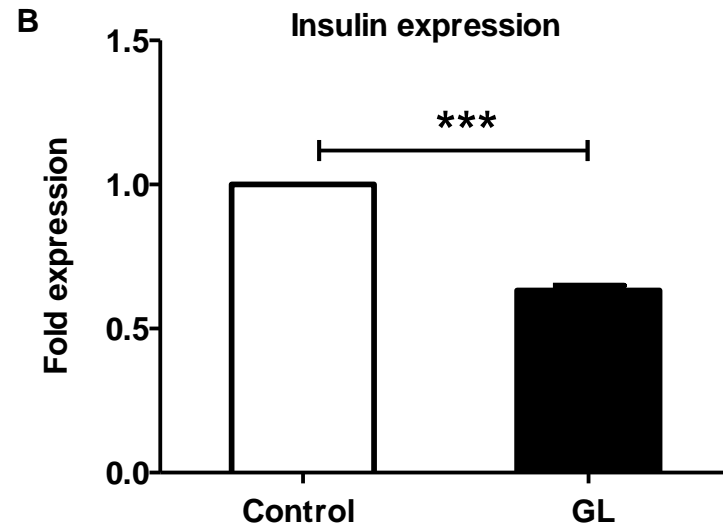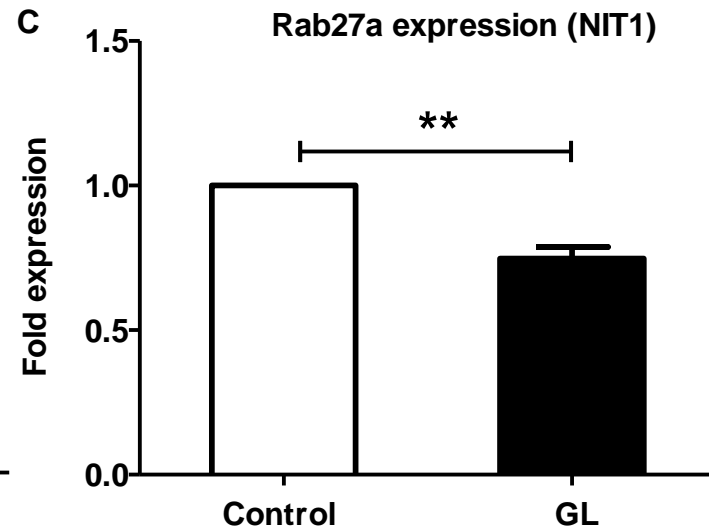

Supplement: Additional file 5: Figure S5 — Chronic GL conditions reduce insulin synthesis and vesicle transport. Rat islets or NIT1 cells were cultured under normal conditions (control) or with 16.7 mM glucose and 500 μM palmitate for 72 h (GL). Post 72 h, RNA was isolated from both islets and cells. The mRNA levels of PDX1 (A) and Insulin (B) were measured in islets and Rab27a levels (C) in cells were measured as described in the materials and methods. Data are expressed as mean±SEM and statistical analysis was performed using the unpaired Student’s t-test. (*P<0.05, **P<0.01 and ***P<0.001, n=4). [file 1471-2121-14-31-S5.pdf]
