# Supplementary material for: Chronic glucolipotoxic conditions in pancreatic islets impair insulin secretion due to dysregulated calcium dynamics, glucose responsiveness and mitochondrial activity
Source: BMC Cell Biol. 2013 Jul 1;14:31. doi: 10.1186/1471-2121-14-31 (PMC3704974; doi:10.1186/1471-2121-14-31)
Supplement: Additional file 6 — Supplementary methods. [file 1471-2121-14-31-S6.doc]

**SUPPLEMENTARY METHODS:**

***Rat islet isolation:***

Male Wistar rats (8 weeks, 180-240gm body weight; Charles River Lab, USA) were used for islet isolation. All experimental protocols have been approved by Institutional Animal Ethics Committee (IAEC), which is recognized by the Committee for the Purpose of Control and Supervision on Experiments on Animals (CPCSEA), India. Animals were killed by excess anesthesia and the pancreas was taken out in a tube containing HBSS (HEPES buffered saline solution) (pH7.4; Sigma). Pancreas/pancreata were washed in the same buffer and cut into 1-2mm pieces. These pieces were then digested by collagenase-II (2mg/ml in HBSS; Sigma) at 37^o^C for 20min. The reaction was stopped by adding two volumes of culture medium (RPMI containing 10%FBS; Invitrogen). The mixture was gently triturated followed by centrifugation at 100X*g* for 5min. The pellet was washed with HBSS (Sigma) and then resuspended in 4ml of Histopaque (1.119 gm/ml; Sigma). Culture medium and 3ml of Histopaque (1.077mg/ml; Sigma) were overlaid on this suspension, followed by density gradient centrifugation. The islets were recovered from the interface of culture medium and Histopaque 1.077 and then washed with HBSS. The purified islets were then handpicked under a stereo-zoom microscope (Nikon, Japan) and used for subsequent experiments.

***Measurement of cellular ROS level***

NIT1 (ATCC) cells were cultured with 5.5mM glucose (control) or 16.7mM glucose and 500μM palmitate (GL) for 72h. After treatments, NIT1 cells were loaded with DCFH-DA dye (Invitrogen) for 1 h to estimate ROS levels. Cells were then lysed and lysates were transferred to 96-black well plate to measure increase in DCF fluorescence at 485nm excitation and 528nm emission. Amount of ROS was normalized to total cellular DNA which was measured using bis-benzamide fluorescence at 360nm exication and 460nm emission.

***Measurement of nitric oxide release***

NIT1 cells were cultured under control or under GL conditions for 72h. The culture medium was harvested followed by centrifugation at 100*xg* for 5min. Clear supernatant (50µl) was mixed with equal amount of 1X Griess reagent in 96-well plate and incubated for 15min and absorbance was read at 540nm. Amount of nitric oxide release was quantified by a standard curve prepared using sodium nitrite. Nitric oxide release was normalized to total cellular proteins estimated by Bradford reagent (Bio-Rad).

***MTT Assay***

NIT1 cells were cultured under control or under GL conditions for 72h. After incubation, MTT dye (3-(4,5-Dimethylthiazol-2-yl)-2,5-diphenyltetrazolium bromide, a yellow tetrazole) was added in culture medium and cells were further incubated for 2.5h. After incubation, reduced form of MTT (formazan) was dissolved in DMSO and amounts were estimated by 560nm absorbance after correction with 640nm absorbance. The amount of MTT reduced to formazan was represented as % of control.

***Caspase-3 assay***

NIT1 cells or rat islets were cultured with 5.5mM glucose (control) or 16.7mM glucose and 500μM palmitate (GL) for 72h. After the incubation period, cells were lysed in lysis buffer followed by estimation of caspase-3 activity by its ability to cleave its substrate (Ac-DEVD-pNA; Sigma) and release of pNA (p-nitroanilide). The pNA released was measured at 405nm absorbance. Activity of caspase-3 (as pico-moles of pNA released/min) was normalized to total cellular protein using Bradford reagent (Bio-Rad).

***NADPH assay***

NIT1 cells were cultured under control or under GL conditions for 72h. After incubation cells were lysed and NADPH levels were measured in total cell lysates using EnzyChrome NADPH assay kit (BioAssy Systems) according to manufacturer’s protocol. Amount of NADPH (nano-moles) was normalized to total cellular protein using Bradford reagent (Bio-Rad).

***Estimation of lactate release***

NIT1 cells were cultured under control or under GL conditions for 72h. After incubation culture medium was collected and centrifuged to remove any debris. Amount of lactate in culture medium was estimated using EnzyChrome NADPH assay kit (BioAssy Systems) according to manufacturer’s protocol. Amount of lactate release (micromoles-moles) was normalized to total cellular protein using Bradford reagent (Bio-Rad).

***Primer sequences used in the study***

The primers used to detect rat gene expression are as follows:

GCKFor (caggacttgcactttcacga) and GCKRev (gcaaggtccaggaagcatt);

PCFor (agggcactatccgaaaggtt) and PCRev (tgttgagaggcttggggtag);

CD36For (atcggaactgtgggctcatt) and CD36Rev (ccgttttcacccagtttttga);

PDX1For (aacgccacacacaaggagaaca) and PDX1Rev (aggaaagagtcccagaggcaga);

INSFor (ttcttctacacacacccaagtcccgt) and INSRev (actgatccacaatgccacgct);

RAB27aFor (tcacctgcagttatgggaca) and RAB27aRev (aggtcgaacagaagcaggaa);

GLUT2For (gtcacaccagcatacacaacacca) and GLUT2Rev (acccaccaaagaatgaggcga);

PPARαFor (aatccacgaagcctacctga) and PPARαRev (gtcttctcagccatgcacaa);

ACTBFor (ctgtgctatgttgccctaga) and ACTBRev (gctcattgccgatagtga);

For measuring metabolic stress markers in the islets, the following primers were used:

IL1betaFor (tgacccatgtgagctgaaag) and IL1betaRev (gggattttgtcgttgcttgt);

NOS2AFor (ggaagaaatgcaggagatgg) and NOS2ARev (tctgcaggatgtcttgaacg);

ACTBFor (ctgtgctatgttgccctaga) and ACTBRev (gctcattgccgatagtga);

The primers used to detect mouse gene expression are as follows:

Rab27A1For (cctgcacctgatcatgaagc) and Rab27A1Rev (ccccttctccttctcctcac);

18S rRNA For (ggacacggacaggattgaca) and 18S rRNA Rev (cgctccaccaactaagaacg)

For measuring mitochondrial DNA copy number in the rat islets, the following primer sequences were used.

Cox1For **(**gcagggatacctcgtcgtta) and Cox1Rev (cggccgtaagtgagatgaat);

HPRTFor **(**cctaagatgagcgcaagttgaa) and HPRTRev (ccacaggactagaacacctgctaa)
